# Incidence of Etiologic Factors in Squamous Cell Carcinoma of Head and Neck in Ahvaz 

**Published:** 2012

**Authors:** Soheila Nikakhlagh, Nader Saki, Mahmood Hekmat shoar, Amin Sartipipor, Sara Saki

**Affiliations:** 1 *Associated Prof. of Otorhinolaryngology of imam Khomeini hospital*; 2 *Resident of Otorhinolaryngology of imam Khomeini hospital*; 3 *General Physician of Ahvaz health care*; 4 *Medical Student of shiraz university*

**Keywords:** Etiologic factors, Head, Neck, Smoking, Squamous cell carcinoma

## Abstract

**Introduction:**

Squamous cell carcinoma (SCC) is the most common head and neck malignancy. Smoking, alcohol consumption, viral infections, exposure, oral hygiene, and dietary, genetic, and occupational factors are the most important etiologic factors. The aim of this study was determining the incidence of etiologic factors in head and neck SCC.

**Materials and Methods:**

This is a cross-sectional survey study for the determination of the etiologic factors of head and neck squamous cell carcinoma over a five-year period in the Otolaryngology Department of the Imam Khomeini & Golestan hospitals in Ahwaz.

**Results:**

176 patients, comprising 151(85.8%) men and 25(14.2%) women, were studied. Overall mean age was 67.2 years. 148 (84.1%) patients were smokers. prolong exposure to chemical fertilizer in 101 (57.4%) patients, Sun exposure in 21 (11.9%) patients, Low socioeconomic status in 124 (70.5%) patients, poor oral hygiene in 128 (72.7%) patients, high intake of hot tea drinking in 84 (47.7%) patients and malignancies in family in 12 (6.8%) patients were the most frequent risk factors. 17 (9.6%) patients have had opioid addiction and HPV was positive in 7 (3.9%) patients by PCR.

**Conclusion:**

According to this study, tobacco smoking was the most important etiologic factor and had a strong effect on risk of head and neck squamous cell carcinoma. Other factors are also important and need more research study.

## Introduction

Cancer has always covered a wide range of issues in medicine, accounting for a large portion of the modern medical research. Despite the undeniable and significant breakthroughs which have helped many cancer patients, identifying the causative factors and fighting them has always been the focus of the attention of researchers. The most frequent questions asked by patients’ relations include: What causes cancer? Do genetic and environmental factors contribute to it? The answers lie in the epidemiology and etiology of the diseaseThe most common environmental contributing factors in head and neck squamous cell carcinoma include: smoking tobacco and drinking (1, 2), exposure to and inhaling chemicals such as hydrocarbons, nickel, and wood dust (3, 5), eating habits (2, 4); poor oral and dental hygiene; ultraviolet radiation and viruses, especially the papilloma virus (3-5, 22).

Nonetheless, the high incidence of these tumors in certain geographical locations, for example hypopharyngeal cancers in Iran, India and Japan (4, 6), made us conduct a study in the city of Ahvaz, located in the Iranian province of Khuzestan, hoping to set a starting point for extensive epidemiologic studies aiming at identifying the causative factors of head and neck cancers in Iran.

## Materials and Methods

In this cross-sectional, descriptive, and prospective research, from September 2006 to December 2010, patients in the ELT and surgical wards of the Imam Khomeini and Golestan hospitals (the major university hospitals in Ahvaz serving cancer patients) who had undergone diagnostic and therapeutic procedures and who were pathologically proven cases of SCC at multiple loci (including lips, mouth, pharynx, tonsils, nose, paranasal sinuses and earlobes) were studied. 

The study subjects included all the inpatients and also those who had not been hospitalized due to extensive metastasis or need for chemotherapy. Interviews and the completion of the questionnaires were directly conducted by the researchers, and all the information was collected based on self-reports.

Variables related to the causative agents of head and neck SCC (including age; sex; occupation; residence; history of the disease; history of tobacco smoking, alcohol consumption and substance abuse; exposure to chemicals; diet and eating habits; oral and dental hygiene; long exposures to sunlight; history of the disease in the family; substance abuse; and HPV infection) were studied.

To investigate HPV infection, the PCR (Polymerase Chain Reaction) method was used on biopsied tumor tissues and exfoliated moth cells from mouthwash samples. To identify the HPV types, the RLBA (Reverse Line Blot Assay) method, specific to 35 types of HPV, was employed. The results were analyzed using SPSS 16 based on descriptive statistics.

## Results

Of the 176 patients studied, 151 patients were male and 25 were female. The average age of the female patients was calculated at 65.7 years and that of the male patients was 67.2 years.

All the patients were married, and as for history of diseases, 38 patients mentioned cardiac, thyroid, anemia and hyperlipidemia conditions.

Twelve patients mentioned histories of malignancy in their immediate relations.

As regards tobacco smoking, 148 out of the 176 patients used tobacco products; 50 of whom smoked over 20 cigarettes a day and others fewer.

The average time of tobacco smoking was 41.8 years.

**Fig 1:**
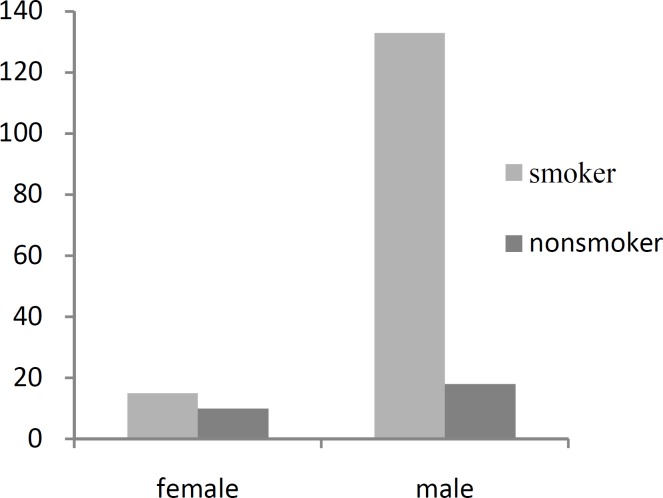
Sex Distribution of Smoking

Interestingly, 15 out of the 25 female patients used tobacco products (Fig 1).

Twenty-four patients used handmade cigarettes, 67 used local cigarettes and 57 used foreign brands.

Only 4 patients mentioned alcohol consumption.

Twenty-four patients had a history of oral lesions, and 101 patients were occupationally exposed to chemicals or chemical fertilizers, the average exposure time estimated at 21 years.

Twenty-one patients (11.9%) were continually exposed to sunlight due to occupational conditions.

One hundred and fourteen patients had a history of drinking hot tea, 43 of whom drank over 1000 CC a day, 41 patients between 500 and 1000 CC, and only 30 patients below 500 CC.

Seventeen patients mentioned substance abuse, of whom 12 used inhaled drugs, two oral and three injections.

Oral and dental hygiene was very poor in 107 cases, poor in 21 cases, and satisfactory in 48 cases. None of the patients had a history of radiotherapy.

Seven cases out of 176 patients (3.97%) tested positive HPV using the PCR method. Three case were type 16, two cases type 18, one case type 57, and one case type 33.

**Fig 2:**
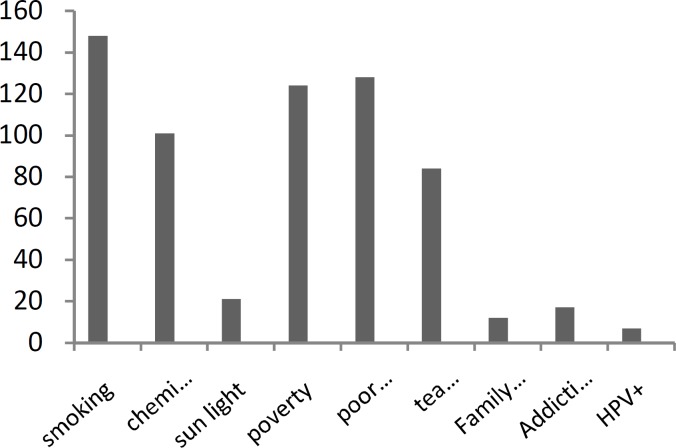
Etiologic factors in squamous cell carcinoma of head and neck

As for the economic status, 72 patients were at a very low economic level (an income of under 150,000 tomans per month), 52 patients were at a low economic level (an income of 150,000 to 300,000 tomans), and the rest were at a good economic level (Fig 2).

**Fig 3:**
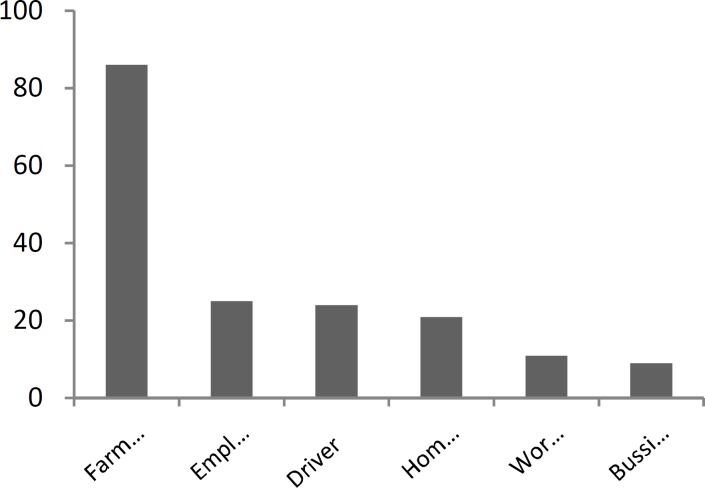
Incidence of Occupational Distribution

The distribution of different occupations in patients is shown in Fig 3.

## Discussion

Malignant tumors of the head and neck are relatively common type of cancer (7), in fact, they comprise some 30-40% of all cancers in some societies such as India (8).

In our study the ratio of the male patients to the female patients was 6 to 1. This ration used to be 15 to 1 in the past, but the incidence in females has gradually risen so that the world’s figures are now at 3.4 to 1 (9-11).

In our study the high tobacco smoking rates (84.1%) in both female and male patients indicate the significant effect of this agent in the etiology of head and neck cancer. In fact, tobacco smoking has had a high rate among different tribes and nomads of the Province of Khuzestan, especially in socially and economically poorer areas. These patients also mentioned other forms of tobacco consumption such as hookah smoking. 

According to our study, 21 patients smoked handmade cigarettes, which naturally contained more impurities and higher toxicity.

In a study by Tryggvason et al, 2009, on the epidemiology of head and neck cancers, they concluded that cigarette smoking and alcohol consumptions were the major risk factors (7).

Dietz, on the other hand, observed in his epidemiologic studies that alcohol was not that carcinogenic when consumed alone, but that it could be pointed out as a contributing factor in those who smoked a lot.

In a study conducted by Razmpa at the Imam Khomeini Hospital, Tehran, Iran, on 100 cases of pharyngeal epithelial cancer, smoking and smoking combined with alcohol consumption were observed as the major risk factors (13).

Other similar studies have mentioned smoking as a major risk factor (8, 11, 14-16)

After tobacco, alcohol is considered one of the most important head and neck SCC risk factors (7, 11, 15), but in this study, only four people reported a history of alcohol consumption, which the author believes to be closely related to the religious and cultural beliefs of the society; basically, alcohol seems to play an insignificant role as a causative agent in cancer in Muslim societies.

The other significant finding was the relationship between occupations conditions and the disease incidence. A significant number of the patients were farmers.

Other occupations included driving, office work, labor, homemaking and shop keeping.

In the examination of the relationship between occupations and cancer agents, UV chemical fertilizer exposure was a significant point; the average time of exposure to chemical fertilizers was calculated at 21 years. 

This calls for more studies and researches.

There have been several studies on occupational factors as predisposing factors in head and neck cancer, having identified such occupational factors as exposure to nickel, chromium, hydrocarbons, asbestos, cement, and wood dust (17).

According to the case histories, there was no history of working with nickel, working in wood industries, dermatological conditions, STDs or schistosomiasis.

The other significant point was the poor economic conditions. This was the case with 70.5% of our patients, which could have been a contributing factor through malnutrition, vitamin deficiency, lack of antioxidant consumption, etc.

Iron deficiency is also noteworthy as a predisposing factor in cancer. The role of dietary factors in oral and hypopharyngeal cancers has been particularly significant in the studies (18, 19).

Poor oral and dental hygiene in most of the patients in this study (72.8%) is the next etiological factor. Other studies have also shown periodontitis, insufficient use of brushing and mouthwash and the loss of teeth as independent variables (20, 21).

The results of our studies does not show a significant relationships between the presence of the human papilloma virus and head and neck cancer.

It seems that there are other predisposing and causative agents than the papilloma virus (such as socioeconomic factors and tobacco smoking) in head and neck SCCs, and that the papilloma virus only has a secondary role (22), which is in direct contrast with the results of the studies conducted in other places in the world, which emphasize the role of HPV as an etiological factor in head and neck SCCs (7, 11, 15, 23).

Destefani et al noted the etiological role of a certain type of tea called mate consumed in the Chemiary region of Brazil. Those who consumed over 1.5 liters of this tea per day were 4.9 times more likely to have cancer(24).Excessive use of tea was a significant point in our study. Twenty-four point four percent of the patients used over one liter of tea per day and 23.3% between 500 and 1000 CC. Studying mate, Destefani pointed out the presence of phenol in it and considered it a carcinogenic solvent.

Are there similar compounds in the tea consumed in Iran? The answer to this question calls for further investigation, including the identification of the most common type of tea consumed in Iran and of the impurities in it.

The studies conducted by Sturgis et al on the likelihood of the role of genetic factors in the etiology of head and neck epithelial cancers (25) has also been observed in our patients. Histories of cancer in immediate relatives was observed in 6.8% of the patients in our study.

## Conclusion

Cancer has always been a major issue in medical research. Even more important is the identification of etiological factors in cancers, which has always been the concern of the patients, their relatives and the medical staff. As with the studies conducted in other parts of the world, tobacco smoking was observed as the most significant etiological factor in our study. The study of other etiological factors will help identify the causative factors, which requires extensive epidemiologic studies.
